# An Innovative Influenza Vaccination Policy: Targeting Last Season's Patients

**DOI:** 10.1371/journal.pcbi.1003643

**Published:** 2014-05-22

**Authors:** Dan Yamin, Arieh Gavious, Eyal Solnik, Nadav Davidovitch, Ran D. Balicer, Alison P. Galvani, Joseph S. Pliskin

**Affiliations:** 1 Department of Epidemiology of Microbial Diseases, Yale University, New Haven, Connecticut, United States of America; 2 Department of Industrial Engineering and Management, Ben Gurion University of the Negev, Beersheba, Israel; 3 Faculty of Business Administration, Ono Academic College, Kiryat Ono, Israel; 4 Department of Health Systems Management, Ben Gurion University of the Negev, Beersheba, Israel; 5 Clalit Research Institute, Clalit Health Services, Tel Aviv, Israel; 6 Department of Health Policy and Management, Harvard School of Public Health, Boston, Massachusetts, United States of America; Duke University, United States of America

## Abstract

Influenza vaccination is the primary approach to prevent influenza annually. WHO/CDC recommendations prioritize vaccinations mainly on the basis of age and co-morbidities, but have never considered influenza infection history of individuals for vaccination targeting. We evaluated such influenza vaccination policies through small-world contact networks simulations. Further, to verify our findings we analyzed, independently, large-scale empirical data of influenza diagnosis from the two largest Health Maintenance Organizations in Israel, together covering more than 74% of the Israeli population. These longitudinal individual-level data include about nine million cases of influenza diagnosed over a decade. Through contact network epidemiology simulations, we found that individuals previously infected with influenza have a disproportionate probability of being highly connected within networks and transmitting to others. Therefore, we showed that prioritizing those previously infected for vaccination would be more effective than a random vaccination policy in reducing infection. The effectiveness of such a policy is robust over a range of epidemiological assumptions, including cross-reactivity between influenza strains conferring partial protection as high as 55%. Empirically, our analysis of the medical records confirms that in every age group, case definition for influenza, clinical diagnosis, and year tested, patients infected in the year prior had a substantially higher risk of becoming infected in the subsequent year. Accordingly, considering individual infection history in targeting and promoting influenza vaccination is predicted to be a highly effective supplement to the current policy. Our approach can also be generalized for other infectious disease, computer viruses, or ecological networks.

## Author Summary

WHO/CDC recommendations prioritize influenza vaccinations primarily on the basis of age co-morbidities, but have never considered targeting vaccination for individuals previously infected with influenza. An individual's infection risk is governed by his or her contacts as manifested by his or her social interactions. Thus, through contact network simulations that capture contact patterns, we show here that individuals previously infected with influenza have a disproportionate probability of being highly connected within networks and thus serve as the super-spreaders. Accordingly, targeting them is effective in curtailing transmission. In addition to social interaction, a variety of factors, including genetics, co-morbidities, demographics, and epidemiological characteristics, may affect the risk and severity of influenza infection. Regardless of whether individuals are predisposed to infection because of these factors, or social interactions, we show that they can be identified through previous infection. Empirically, our analysis of medical records of influenza diagnosed in both hospitals and clinics confirms that in every age group, case definition for influenza, clinical diagnosis, and year tested, patients infected in the year prior had a substantially higher risk of becoming infected in the subsequent year. Thus, considering individual infection history in targeting and promoting influenza vaccination is predicted to be a highly effective supplement to the current prioritizations as it focuses on people with greater risk to become infected and transmit.

## Introduction

Influenza has a long history of causing substantial morbidity, mortality and economic losses annually [1]–[3]. In Israel, influenza is responsible for about 801,200 reported infections (around 10% of the population), 4130 hospitalizations, 1140 deaths, and economic costs of 261 million dollars [3], [4], while in the US, influenza is responsible for 610,600 life years lost and economic loss of $87.1 billion annually [1]. Influenza vaccination is the primary approach to reduce the disease burden and is important not only for those vaccinated, but also to reduce transmission [2]. Recommendations by the World Health Organizations (WHO) [5], the U.S. Center for Disease Control and Prevention (CDC) [2], as well as the Israeli Ministry of Health have prioritized vaccination based on age, profession, and co-morbidities. However, these recommendations have not considered individual influenza infection history as an indication of future risk that can be used to supplement current policies.

An individual's infection risk is governed by their contacts as manifested by their social interactions. A contact network model captures the patterns of interactions that expose individuals to potential transmission. In the context of contact network epidemiology, centrals, individuals characterized by higher connectivity than average, are more likely both to become infected and to transmit infection [6], [7]. Thus, prioritizing the vaccination of centrals could be effective in curtailing influenza transmission by reducing the network connectivity. However, identifying centrals is challenging [8], because the contact network is generally unknown.

One study [6] offered a novel way to reach the centrals in a network by randomly choosing individuals and asking them to deliver a vaccination dose to one of their contacts, an approach known as the *‘acquaintance immunization strategy’*, suggests an indirect way to locate the centrals. Although this approach is an effective way to curtail transmission in both computer and population networks, it would be challenging to implement such a policy in the case of influenza vaccination.

In the current study we offer a practical way to devise a vaccination policy using the simple logic of targeting previous influenza patients. We propose that even in the absence of information about network structure, centrals are at higher risk of influenza infection and can thus be identified as being disproportionately represented in the pool of individuals who were previously infected. Further, in addition to social interaction, a variety of factors, such as genetics, co-morbidities, demographics, and epidemiological characteristics [2], may affect the risk and severity of infection, and remain relatively invariable over time. Regardless of whether individuals are predisposed to infection because of these factors or contact connectedness, they can be identified through previous infection. This approach is more straightforward than attempting to target individuals based on all possible risk factors, particularly as some risk factors may be unknown, difficult to identify or politically challenging to implement. Although the effectiveness of a policy that targets those previously infected initially seems to be counter-intuitive, since previously infected are likely to have partial protection against subsequent infection due to cross-reactivate antibodies [9], we found that previously infected individuals are much more likely to be infected even when taking into account biologically realistic rates of cross-reactivity [10].

Our findings are based on contact network epidemiology simulations and confirmed by empirical clinical data provided by the two largest Health Maintenance Organizations (HMOs) in Israel, covering more than 74% of the Israeli population. Our study is the first to address the interplay among previous infection history, immunological cross-reactivity, and social behavior as the basis for an innovative yet feasible supplement to the current influenza vaccination policies.

## Methods

### Ethics statement

The surveillance data were analyzed anonymously, and approved to be used by the Clalit health services sub-Helsinky institutional review board, signed and approved by Dr. Eitan Wertheim, protocol number 127/2012.

### Contact network simulations

Our simulations were applied to an epidemiological contact network based on the Portland population [11]. The Portland contact network derives from detailed microscopic simulation-based modeling and integration techniques performed by the Network Dynamic and Simulation Science Laboratory (NDSSL) at Virginia Tech with the purpose of creating a contact network reflecting an urban population [11]. The network includes 1,575,861 nodes, each of which represent an individual and 19,681,820 edges, each of which represents a contact between individuals.

To determine the robustness of our results, we validated our findings on three alternative small-world [12] *scale-free* networks: the Brightkite location-based network, the Gowalla location-based network, and the Barabási algorithm based network [13]–[15]. These networks vary in terms of the number of contacts, clustering coefficients, and the node-to-node distance [12]. The Brightkite location-based network is based on service providers where users share their locations by checking-in. The network was generated by the Stanford Network Analysis Project using their public Application Programming Interface, which consists of 58,228 nodes and 214,078 edges [16]. The Gowalla location-based contact network is a website where users share their locations by checking-in. The network was collected by the Stanford Network Analysis. It consists of 196,591 nodes and 950,327 edges [13]. We also created a network with 100,000 nodes and 400,000 edges according to the Barabási algorithm [14].

To evaluate centrality for each node in the networks, we calculated two common measures: number of contacts and K-shell decomposition values (K-shell) (Figure S1). Compared with the straightforward number of contacts measure, K-shell also take into account the global connectivity of the nodes to which a node is connected [17], [18].

We used the Susceptible-Infectious-Recovered (SIR) compartmental model [19] to evaluate disease spread within the networks. According to each network configuration, an individual may infect only susceptible neighbors (i.e., nodes with whom they have edges). Given that not all individuals will be susceptible in the beginning of each season [10], [20], [21], we parameterized transmissibility using the effective reproductive number, *R_e_* [21]–[23], defined as the average number of secondary infections resulting from each infective person [19] (Table 1). Protection following infection may last longer than a year [10] due to cross-reactivity between years. We considered the entire possible range of cross-reactivity from 0 to 100%, where 0% corresponds to no immunological protection and 100% corresponds to full protection acquired from influenza infection in the prior year. Depending on vaccine efficacy (Table 1), we assume that a proportion of individuals who are vaccinated are protected for one season [24].

**Table 1:** Parameter ranges and values for numerical simulations.

Symbol	Definition	Distribution/range checked	References
*i_0_*	Initial infection fraction	Uniform(0.0001,0.001)	[23]
*R_e_*	Effective reproductive ratio	1.2–1.6	[23], [26], [31]
*V*	Vaccination rate	0–0.4	[35]
*R*	Vaccination efficacy	0.5–0.8	[2]
*D*	Infection duration (in days)	Normal(3.8, 2)	[22], [36]
*θ*	Cross-reactivity rate	0–1	
*T*	Daily susceptibility rate between two neighbors	Uniform (0.012,0.087)	Supplements

We ran over one million simulations drawing parameter values from distributions that span a biologically realistic range (Table 1), as well as different vaccination rates and efficacies (Text S1). To determine whether previously infected individuals are more likely to be centrals, we evaluated the decile of the centrality score for each node in the network (based on the two measurements of centrality) and the risk ratio of becoming infected for a range of reproductive ratios and for different levels of cross-reactivity compared with the risk of a random individual. In each iteration of the simulation, we ran two successive influenza seasons. In the first season, we randomly vaccinated 0–40% of the population. In the second season of each simulation, we considered three policies: a Random Vaccination Policy (*RVP*), an acquaintance immunization policy *(AIP)*, which targets the acquaintances of a random node [6], and a vaccination policy prioritizing those who were infected in the previous season, Previously Infected Policy (*PIP*). For the *PIP* strategy, we assumed previously infected individuals are vaccinated first. If any vaccine doses remain after those prioritized have been vaccinated, the remaining doses are randomly distributed to the rest of the population.

### Empirical demonstration

Our primary data were provided by Clalit Health Services and Maccabi Health Services and included demographic, socio-economic, and ethnic data [25]. Clalit is the largest HMO in Israel, with membership varying between 3.47–3.72 million, and constituting about 53% of the Israeli population during the 2003–2012 the study period.

The Clalit dataset includes codes of the diagnosis recorded by the physicians in clinics according to the ICD9 protocol (codes 487 for ‘influenza’ and 465 for ‘acute upper respiratory infections of multiple or unspecified sites’) as well as hospitalizations due to influenza or pneumonia (ICD9, 486 ‘pneumonia organism unspecified’ as well as codes 487, 465). Maccabi is the second largest HMO in Israel with 1.4–1.8 million members, constituting about 23% of the Israeli population, during the study period. Their records include full datasets of 12 years from September 1998 to April 2010 with 380,000 records. The data included influenza cases diagnosed in clinics according to the ICD10 protocol (code J11). Our case definition for influenza-like-illness (ILI) in both of the health maintenance organizations is detailed in Text S2.

As influenza infection rates depend on age, we stratified our data analysis by: 0–5, 6–15, 16–25, 26–35, 36–49, 50+. This division also facilitates evaluating the age-specific prioritization of the recommendations of the U.S. CDC as well as the Israeli Health Ministry which currently focus on ages 0–5 and individuals above age 50. In addition, we considered the relative risk of age group 25–35 which, along with children, are disproportionately responsible for transmission [26].

Rather than a case of influenza, an ILI infection might indicate elevated risk for influenza, because transmission routes of many upper respiratory diseases are similar. Thus, an ILI might serve as a predictor of elevated risk for both ILI and actual influenza. For each age group, in both HMOs, we calculated the relative risk of infection for those previously diagnosed with ILI compared with others in the same age group that had not been diagnosed in prior season.

In the Clalit dataset we stratified our ten seasons of data into eleven pairs of two consecutive seasons and calculated the risk of outpatient influenza in season *i* for influenza outpatient patients diagnosed in season *i−1*, the risk of outpatient influenza in season *i* for patients hospitalized in season *i−1*, the risk of becoming hospitalized with influenza in season *i* for outpatient patients diagnosed in season *i−1*, and the risk of becoming hospitalized with influenza in season *i* for patients hospitalized in season *i−1*. In the Maccabi dataset, we stratified our twelve seasons of data into eleven pairs of two consecutive seasons and calculated the risk of outpatient influenza in season *i* for influenza outpatient patients diagnosed in season *i−1* (Text S2).

Not all influenza patients seek medical treatment [1], [27], and some people might have higher tendency to seek medical treatment when infected with influenza than others, potentially leading to an overestimation relative risks. In addition, individuals are likely to visit the same physician when infected with influenza, and the latter might not diagnosis the infection as ILI. Thus, we compared evaluations of the policies under the conservative assumption that individuals can be divided into those that either seek medical treatment when infected, or not seek medical treatment. Under this conservative assumption, we calculated an adjusted relative risk of outpatient infection by removing in each age group members who were never an outpatient along the entire period tested.

## Results

The risk of future influenza infections for individuals is determined by the interplay between two countering factors: social interaction, which governs exposure probability, and cross-reactivity protection acquired from previous infection. We found that these factors will affect: 1) the conditions under which central individuals have a higher risk of infection than non-centrals, 2) the conditions under which individuals infected in the prior season have a higher risk to become infected in a succeeding season, and, consequently, 3) the conditions in which the targeting of last season's patients for influenza vaccination will serve as an effective policy to decrease morbidity. Overall, our analysis demonstrates that social interaction dominated in determining the overall effectiveness of targeting influenza vaccination to previously infected individuals in vast ranges of realistic conditions, as elaborated below.

**Figure 1:**
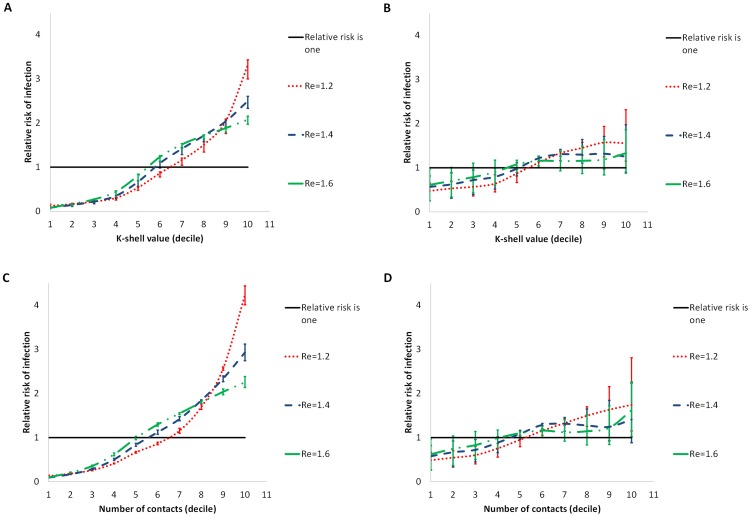
The relative risk of infection given parameters of centrality. The mean and 95% confidence interval of relative risk of infection for an individual compared to the rest of the population, given his/her K-shell (panels A and B), and number of contacts (panels C and D) for cross-reactivity levels of 0% (panels A and C) and 80% (panels B and D) for effective reproductive number, *R_e_* = 1.2 (dotted red), 1.4 (dashed blue) and 1.6 (dot-dashed green). A relative risk above one represents higher risk of infection, compared with the rest of the population.

**Figure 2:**
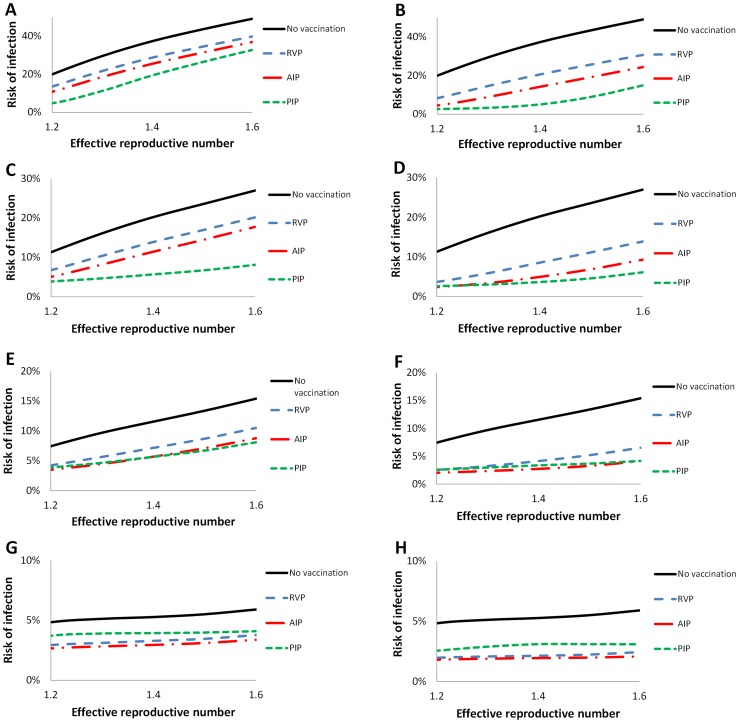
Mean risk of infection following vaccination. The mean risk of infection evaluated over the parameters ranges in Table 1 for *RVP* (dashed blue), *AIP* (dashed-doted red), *PIP* (dashed green), as well as no vaccination (solid black), for cross-reactivity levels of A and B) 0%, C and D) 40%, E and F) 60% G and H) 80%. In the second season, for *RVP*, *AIP* and *PIP* strategies, vaccination coverage for A, C, E and G) 15% and for B, D, F and H) 30%, and vaccine efficacy of 75%. *PIP* is preferable than *RVP* in reducing morbidity for panels A–F, and more preferable than *AIP* for panels A–D. As explained in the main text, the risk of infection decreases as the cross-reactivity increases.

Our contact network simulations reveal that individuals infected with influenza in the prior year have a greater probability of being more central than others. This finding is robust to the two different measurements of centrality considered (Figures 1 and S2), and over the range of biologically realistic epidemiological parameters for influenza (Table 1). The results were more sensitive to the initial proportion of susceptibles. Specifically, the greater the cross-reactivity and attack rate are in the previous season, the lower is the risk of infection for the central individuals relative to the rest of the network. For example, in the Portland network, we found that individuals with higher K-shell values or higher number of contacts than others have a higher risk of infection when cross-reactivity ranges between 0–60%. For cross-reactivity higher than 60%, and particularly for the upper estimates of influenza transmissibility (i.e., *R_e_≥1.4*), individuals in the ninth or tenth decile with the highest K-shell value or number of contacts are less likely to get infected relative to individuals in the fifth decile, because the former were more likely to have cross-protection from previous exposure. Nevertheless, even in this case, individuals who have a centrality score above the median have a higher risk for re-infection than individuals below the median (Figures 1 and 2). Collectively, centrals are disproportionately represented in those who were previously infected even when cross-reactivity reached 80%.

For seasons between which there is no cross-reactivity, we showed analytically that prior influenza patients have a higher risk of infection in the succeeding season (text S1). Consistent with this analytical finding, our simulations on the Portland network also demonstrate that when there is no cross-reactivity, the relative risk of previous patients ranges from 2.3–3.1 compared to individuals not infected in the prior season. However, this risk can also fall below one in cross-reactivity rates above 60% (Figure S3).

**Figure 3:**
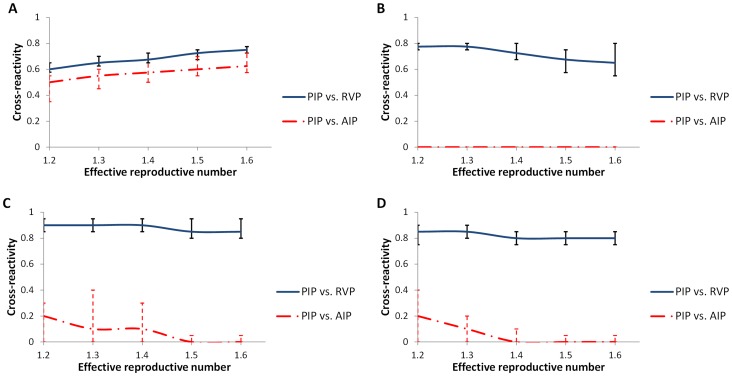
Mean indifference curves for *PIP* vs. *RVP* and *PIP* vs. *AIP*. The curves are shown as a function of the effective reproductive number and cross-reactivity for A) the Portland Network B) Barabási algorithm-based network C) Brightkite Network D) Gowalla Network. Above each curve, *RVP/AIP* is the recommended policy, whereas below the curve, *PIP* is recommended.

We compared three policies: a Random Vaccination Policy (*RVP*), the acquaintance immunization policy *(AIP)*, and a vaccination policy prioritizing those who were infected in the previous season, Previously Infected Policy (*PIP*) (see methods). Overall, in the Portland network, results demonstrate that *PIP* is more effective than *AIP* in reducing morbidity rates even in cases where cross-reactivity is as high as 36–72%. *PIP* is also more preferable than *RVP* even in cases where cross-reactivity is as high as 57–78% (Figures 3, Figure S4 and Figure S5). For example, when cross-reactivity of 40% has been observed and 15% are vaccinated the mean risk of infection would be 14%, 11.5% and 5.6% for *RVP*, *AIP* and *PIP*, respectively; whereas when 30% are vaccinated the mean risk would be 8.6%, 4.6%, and 3.6% for *RVP*, *AIP* and *PIP*, respectively.

The variability observed in our simulations (Figure 3, Figure S4 and Figure S5) arises from the sensitivity of *PIP* to two drivers. The first driver is the vaccination coverage in the previous season or infected in the previous season and who remained protected due to cross-reactivity as those individuals cannot be detected by our policy. The second driver is the incidence in the previous season. If the prior incidence is relatively low, all of those who had been infected in the prior year would be vaccinated in the subsequent year, but the remaining doses will be randomly distributed to the rest of the population, thereby making our policy more similar to *RVP*.

To verify the robustness of our results, we determined the effectiveness of *PIP* for four networks across a range of biologically realistic epidemiological parameters for influenza (Table 1). *PIP* was found to be more effective than *RVP* when cross-reactivity was as high as 57–75% in the Portland Network, 55–80% in the Barabási algorithm-based network, 80–95% in the Brightkite Network, and 75–90% in the Gowalla Network. In comparison with *AIP*, *PIP* was effective in lower values of cross-reactivity levels, ranged from 35–72.5% in Portland Network, 0–40% Brightkite Network, and 0–40% in the Gowalla Network (Figure 3). *PIP* was less effective than *AIP* in the Barabási algorithm based network, possibly as a result of the homogeneity of K-shell values among nodes in this network (Figures S1).

### Empirical demonstration

Given that influenza attack rate ranges epidemiologically between 5–15% [2], our case definition for influenza may be under-reported in the Maccabi dataset and over-reported in the Clalit dataset. Nevertheless, our analysis of over nine million medical records showed that patients diagnosed with ILI in the previous season have a substantially higher risk to be diagnosed with ILI in the succeeding year (Table 2). Excepting children below five who had a lower risk of being hospitalized, diagnosed with outpatient influenza in the season prior, our results were robust for both HMOs, for each age group and for almost every year tested. For example, in the Maccabi dataset within the age group of 35–49, only 1.45% of the population was outpatient ILI. However, for individuals within this age group who were diagnosed in the previous year, the risk of becoming infected in the subsequent year was 11.35%. In the Clalit dataset, within the age group 35–49, 9.36% were outpatient ILI. The risk was about four times higher in patients who were outpatient ILI, and two fold in patients previously hospitalized compared with patients who did not seek medical treatment of ILI in the year prior (Table 2).

**Table 2:** Means risk and relative risk of infection stratified by infection history and age group over the years tested.

Health maintenance organization	Clinical outcome	Population by age	0–4	5–14	15–24	25–34	35–49	>50
**Clalit Health Services**	**Outpatient visits**	**Risk for entire population**	26.36%	11.5%	9.48%	10.44%	9.36%	7.91%
		**Risk given outpatient last year**	44.2%	31.09%	26.91%	27.26%	28.83%	24.89%
		**Risk given hospitalized last year**	29.41%	16.24%	14.27%	14.02%	12.77%	7.38%
		**Relative risk given outpatient last year**	2.32 (1.94–2.62)	3.62 (2.90–4.02)	3.59 (3.03–3.89)	3.36 (3.07–3.69)	4.07 (3.71–4.52)	3.93 (3.59–4.45)
		**Relative risk given hospitalized last year**	1.44 (1.25–1.62)	1.76 (1.43–2.3)	1.86 (1.57–2.10)	1.60 (1.39–1.80)	1.93 (1.57–2.25)	1.12 (0.90–1.27)
		**Adjusted relative risk given outpatient last year**	2.21 (1.91–2.49)	2.36 (2.06–2.61)	2.10 (1.89–2.25)	2.07 (1.88–2.29)	2.24 (2.08–2.52)	1.98 (1.75–2.26)
		**Adjusted relative risk given hospitalized last year**	1.37 (1.22–1.56)	1.15 (1.02–1.56)	1.09 (0.91–1.22)	1.01 (0.85–1.21)	1.06 (0.90–1.21)	0.56 (0.48–0.62)
	**Hospitalizations**	**Risk for entire population**	0.39%	0.06%	0.03%	0.04%	0.07%	0.32%
		**Risk given outpatient last year**	0.31%	0.08%	0.13%	0.05%	0.09%	0.45%
		**Risk given hospitalized last year**	3.22%	3.60%	4.24%	3.23%	3.85%	7.21%
		**Relative risk given outpatient last year**	0.71 (0.53–0.77)	1.33 (1.02–1.64)	7.04 (4.01–12)	1.14 (0.93–1.41)	1.42 (1.17–1.73)	1.42 (1.31–1.54)
		**Relative risk given hospitalized last year**	8.02 (6.58–10.50)	66.6 (47.9–84.8)	219.42 (76.4–564.6)	82.7 (62.1–97.9)	70.13 (42.5–114.4)	25.48 (22.68–31.05)
**Maccabi Health Services**	**Outpatient visits**	**Risk for entire population**	2.04%	2.01%	1.35%	1.58%	1.45%	0.93%
		**Risk given outpatient last year**	10.12%	9.02%	8.33%	10.28%	11.35%	13.77%
		**Relative risk**	5.69 (4.31–7.51)	5.13 (3.06–6.77)	7.09 (3.75–10.30)	7.47 (5.65–10.5)	8.81 (6.21–12.16)	17.11 (11.41–26.12)
		**Adjusted relative risk**	1.32 (0.96–1.68)	1.10 (0.84–1.46)	1.26 (0.89–1.83)	1.27 (0.94–1.74)	1.40 (1.17–1.92)	1.61 (1.48–2.46)

The annual minimum and maximum values observed in the years are presented in parentheses. The relative risk was calculated compared with individuals from the same HMO that sought medical treatment in the year prior. The adjusted relative risk included as members only individuals who were diagnosed as influenza patients at least once along the entire period evaluated (2003–2012 in Clalit dataset and 1998–2010 in Maccabi dataset).

Even when we calculated an adjusted relative risk of outpatient influenza by considering only members who were diagnosed with outpatient influenza at least once in the study period (i.e. 2003–2012 in Clalit dataset and 1998–2010 in Maccabi dataset), the relative risk for individuals previously outpatient was still higher than one in each age group (Table 2). This finding demonstrates that, in addition to age, an individual's infection history plays an important role in determining their subsequent risk of infection.

## Discussion

Our work shows that considering individual infection history in targeting and promoting influenza vaccination would be an effective supplement to the current policy which prioritizes individuals on the basis of age and co-morbidities. Our findings highlight the fundamental role that an individual's social behavior plays in disease transmission [28], and reveals that in the interplay between cross-reactivity and individual risk factors the latter dominates in determining the overall effectiveness of targeting influenza vaccination to individuals infected in the prior season.

Our simulations demonstrate that targeting individuals previously diagnosed with influenza can be effective even if cross-reactivity is as high as 55–80%. Empirical studies suggest that cross-reactivity for a specific type of influenza is typically below or within this range [10]. Additionally, there can be two or three sub-types of influenza circulating within a single season [29] with dominance shifting among sub-types between successive years.

Surveillance systems monitor reporting rates rather than actual prevalence, and thus also include misdiagnoses. Nonetheless, ILI diagnoses, even if from a different etiology, might indicate elevated risk of future infection with influenza, because transmission routes and associated risk factors of many upper respiratory diseases are similar. Given that respiratory infections other than influenza will not elicit cross-reactive antibodies, these misdiagnosed individuals may be at even higher risk for future influenza infection than those who were previously infected with influenza. To evaluate the increased risk of those who were previously infected with influenza versus other respiratory infections, future research should stratify between clinical diagnosis and laboratory confirmation.

While social interactions modeled in our contact network simulations are fundamental to influenza transmission, other factors including genetics, co-morbidities, and demography, contribute to determine risk for an individual. Similar to social tendencies, these other factors will also remain relatively invariable for an individual from year to year. Consequently, previous, current, and future infection risk can be even more effectively predicted by prior infection than what we conservatively estimated from social interaction alone.

If *PIP* is implemented every influenza season, individuals with high connectivity might be targeted in the first year, and therefore will reduce their risk of infection in the subsequent year. However, as a result of this reduced risk, in the third year from initiating the *PIP* policy, they will be less likely to be infected and subsequently targeted. Thus, future studies could evaluate the marginal benefit of considering infection and vaccination history of individuals over several seasons relative to the prior season alone in determining vaccine targets.

Targeting previously infected individuals is a relatively straightforward approach to implement in an HMO system with electronic medical records. For instances, individuals previously infected could be flagged within the electronic records for vaccination targeting by mailing pamphlets, telephone reminders or physician recommendations, practices shown to be effective in promoting influenza vaccination [2]. The suggested policy could reach several sub-populations, such as those based on socio-economic status and ethnicity, that correlated with vaccine uptake and infection rates [30], but which have not been prioritized in the past due to ethical or political reasons. Furthermore, a high level of public adherence to this targeted strategy is likely to be achievable, given that individuals who were recently ill with influenza will probably be responsive to strategies that reduce their personal risk, as has been shown to be a primary motivator in vaccination decisions [23], [31], [32].

We demonstrated the potential benefit of targeting last season's patients for influenza, but such policy may also be applicable to other diseases including respiratory syncytial virus, pneumococcal infections and malaria, for which re-infection is common. Our approach may be generalized to networks outside the public health field, such as ecology and computer science. For example, our approach may determine which computers should be prioritized for antivirus software installations. In fact, a previous study on computer networks showed that computers that were attacked in one simulation run are most prone to attack in other simulation runs [33]. The authors even suggested little variation in the number of reinfections experienced by the same computer in different simulation studies, making our approach likely to be highly effective. In another example, our approach may also be helpful in ecological networks to identify and invest efforts to protect species with most essential to community stability [34].

In summary, we modeled the interplay among previous infection history, immunological cross-reactivity, and social behavior as the basis to generate an innovative influenza vaccination policy. Through contact network simulations we showed that individuals infected in the year prior have higher connectivity in the network, and subsequently increased risk of infection and transmission. Empirically, our analysis of the medical records confirms that in every age group, case definition for influenza, clinical diagnosis and year tested, patients infected in the year prior had a substantially higher risk of becoming infected in the subsequent year. Accordingly, the targeting of individuals infected in the prior year is predicted to be a highly effective supplement to the current policy.

## Supporting Information

Figure S1 **Distribution of measurements of centrality.** The distribution of 1) Number of contacts, and 2) K-shell is shown for A) the Portland Network, B) Brightkite Network, C) Gowalla Network, D) Barabási Algorithm-Based Network.
(TIF)

Figure S2 **Relative risk of infection given parameters of centrality.** The mean and 95% confidence interval of the relative risk of infection for an individual compared to the rest of the population, given his/her K-shell (panels A and B), and number of contacts (panels C and D) for cross-reactivity levels of A) 20% B) 40% C) 60% for effective reproductive number, *R_e_* = 1.2 (dotted red), 1.4 (dashed blue) and 1.6 (dot-dashed green). A relative risk above one represents higher risk of infection, compared with the rest of the population. The figure complements Figure 1.
(TIF)

Figure S3 **Effects of previous illness on future infection proportion.** The mean and 95% confidence interval of the relative risk of infection in individuals previously infected versus individuals not previously infected depending on *R_e_* and cross-reactivity. The black line represents relative risk equal to one.
(TIF)

Figure S4 **Boxplot risk of infection following vaccination.** Box-and-Whisker plots of the difference between the risk of infections for *RVP* and *PIP*, and *AIP* and *PIP*, over the parameters ranges in Table 1, for cross-reactivity of A) 0%, B) 40%, C) 60%, and D) 80%, assuming vaccination coverage of 15% and vaccine efficacy of 75%. This figure corresponds to Figure 2 panels A, C, E, and F.
(TIF)

Figure S5 **Boxplot risk of infection following vaccination.** Box-and-Whisker plots of the difference between the risk of infections for *RVP* and *PIP*, and *AIP* and *PIP*, over the parameters ranges in Table 1, for cross-reactivity of A) 0%, B) 40%, C) 60%, and D) 80%, assuming vaccination coverage of 30% and vaccine efficacy of 75%. This figure corresponds to Figure 2 panels B, D, F, and G.
(TIF)

Figure S6 **Frequency of ILI diagnosis.** The right axis refers to influenza and pneumonia diagnosed in hospitals. The left axis refers to influenza diagnosis.
(TIF)

Text S1 **Supporting information for contact network analysis.**
(DOCX)

Text S2 **Supporting information for data analysis.**
(DOCX)
