# Supplementary figures and images for: An Innovative Influenza Vaccination Policy: Targeting Last Season's Patients
Source: PLoS Comput Biol. 2014 May 22;10(5):e1003643. doi: 10.1371/journal.pcbi.1003643 (PMC4031061; doi:10.1371/journal.pcbi.1003643)

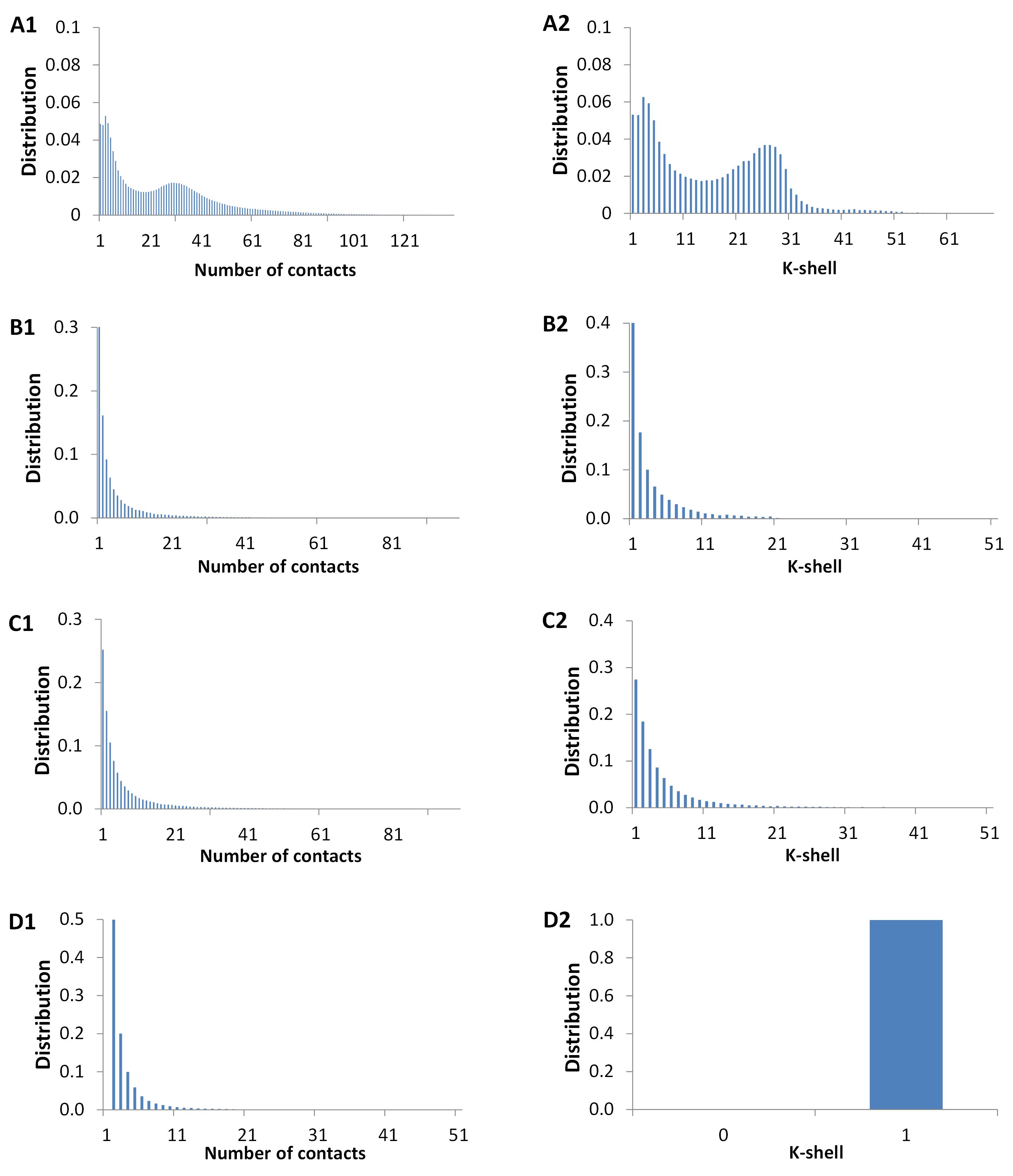

Supplement: Figure S1 — Distribution of measurements of centrality. The distribution of 1) Number of contacts, and 2) K-shell is shown for A) the Portland Network, B) Brightkite Network, C) Gowalla Network, D) Barabási Algorithm-Based Network. (TIF) [file pcbi.1003643.s001.tif]

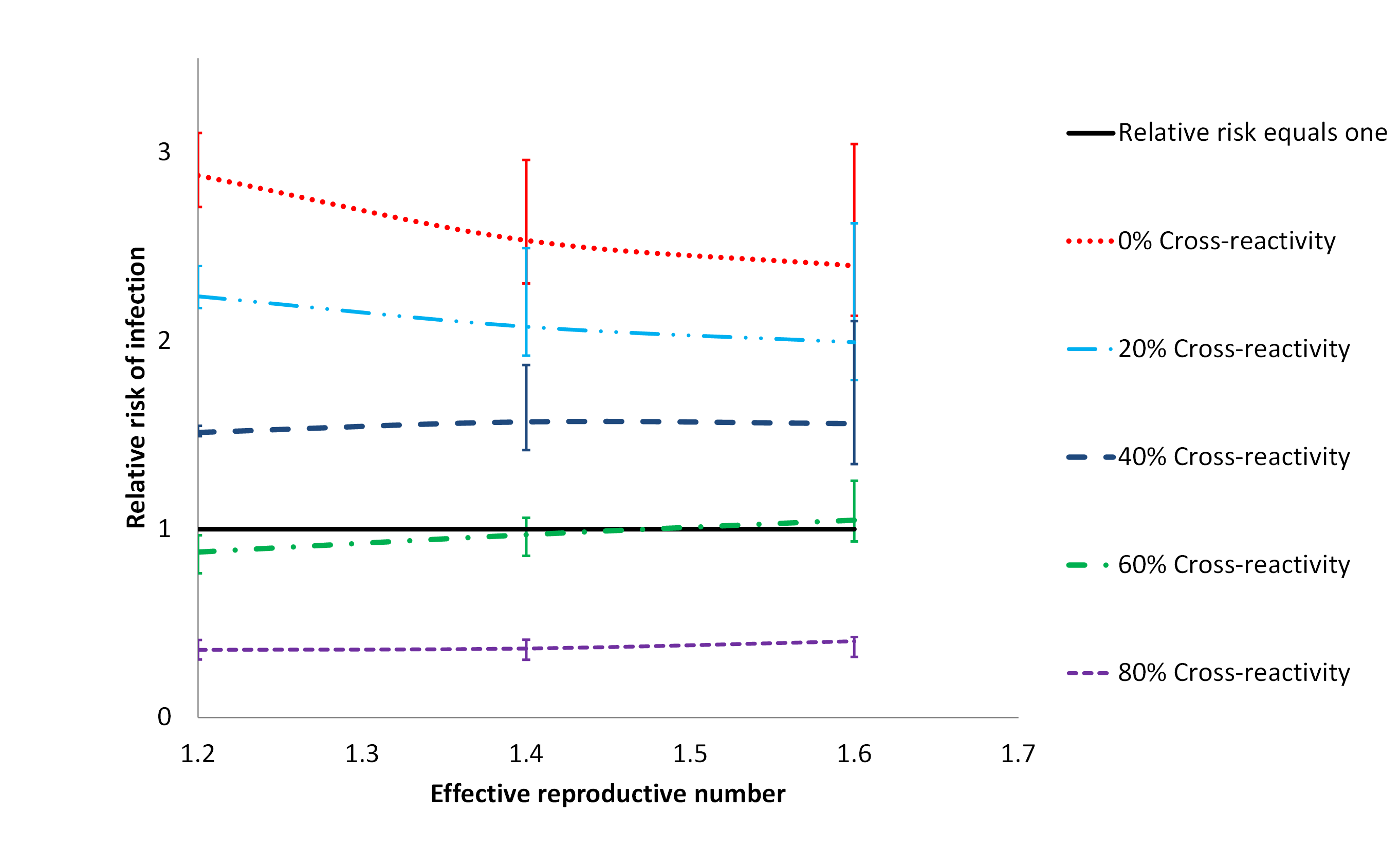

Supplement: Figure S2 — Relative risk of infection given parameters of centrality. The mean and 95% confidence interval of the relative risk of infection for an individual compared to the rest of the population, given his/her K-shell (panels A and B), and number of contacts (panels C and D) for cross-reactivity levels of A) 20% B) 40% C) 60% for effective reproductive number, Re = 1.2 (dotted red), 1.4 (dashed blue) and 1.6 (dot-dashed green). A relative risk above one represents higher risk of infection, compared with the rest of the population. The figure complements Figure 1. (TIF) [file pcbi.1003643.s002.tif]

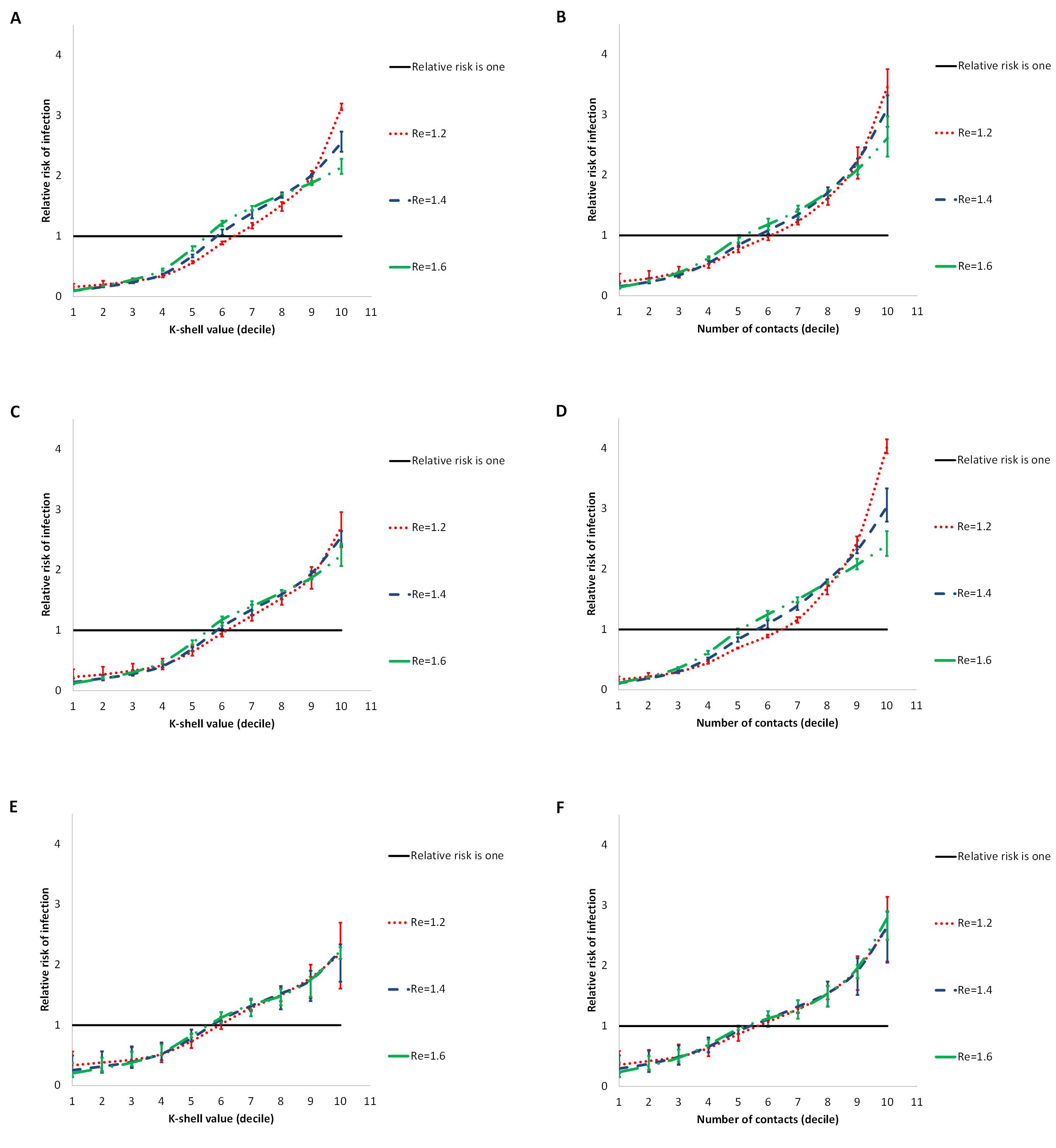

Supplement: Figure S3 — Effects of previous illness on future infection proportion. The mean and 95% confidence interval of the relative risk of infection in individuals previously infected versus individuals not previously infected depending on Re and cross-reactivity. The black line represents relative risk equal to one. (TIF) [file pcbi.1003643.s003.tif]

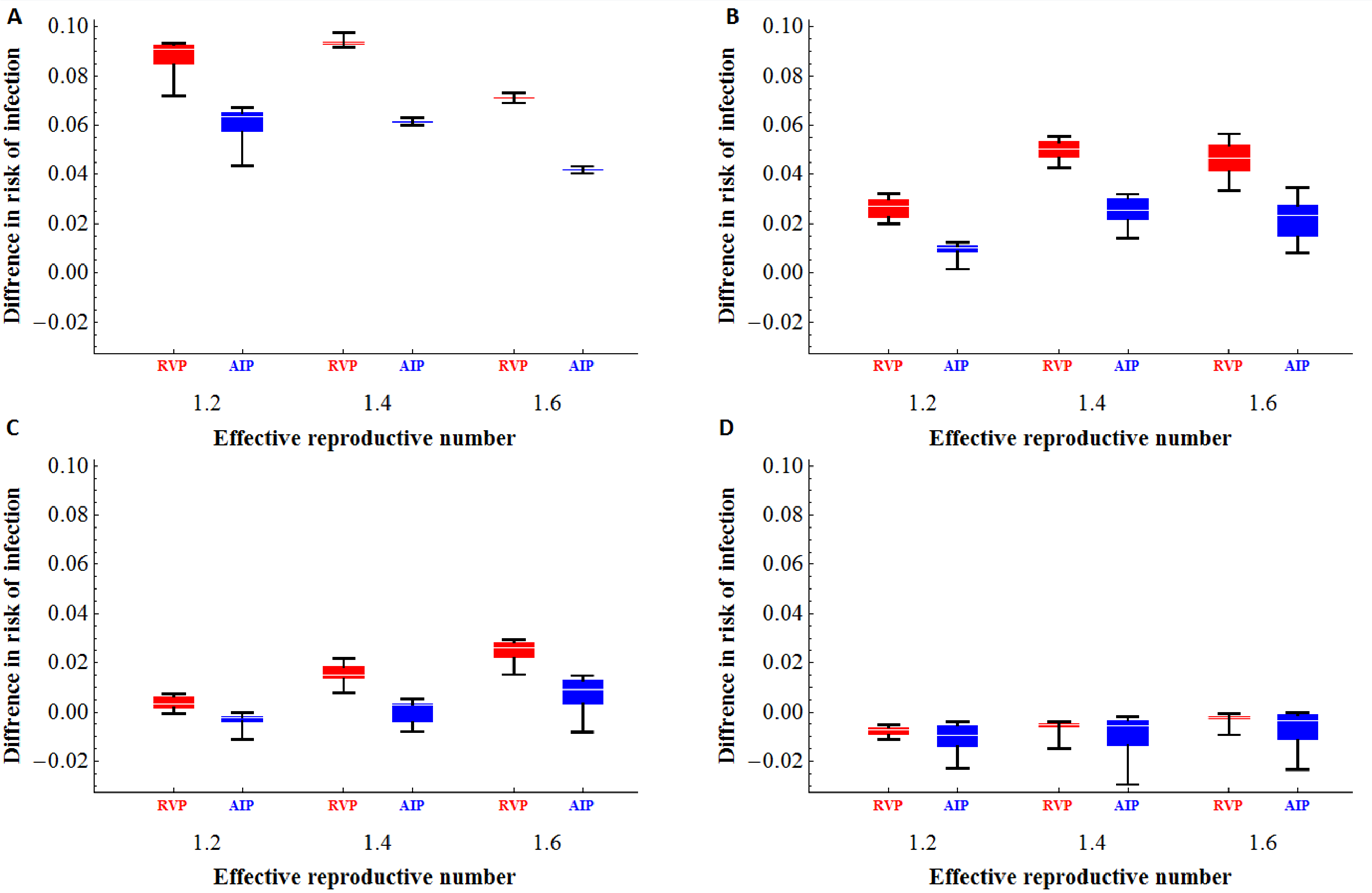

Supplement: Figure S4 — Boxplot risk of infection following vaccination. Box-and-Whisker plots of the difference between the risk of infections for RVP and PIP, and AIP and PIP, over the parameters ranges in Table 1, for cross-reactivity of A) 0%, B) 40%, C) 60%, and D) 80%, assuming vaccination coverage of 15% and vaccine efficacy of 75%. This figure corresponds to Figure 2 panels A, C, E, and F. (TIF) [file pcbi.1003643.s004.tif]

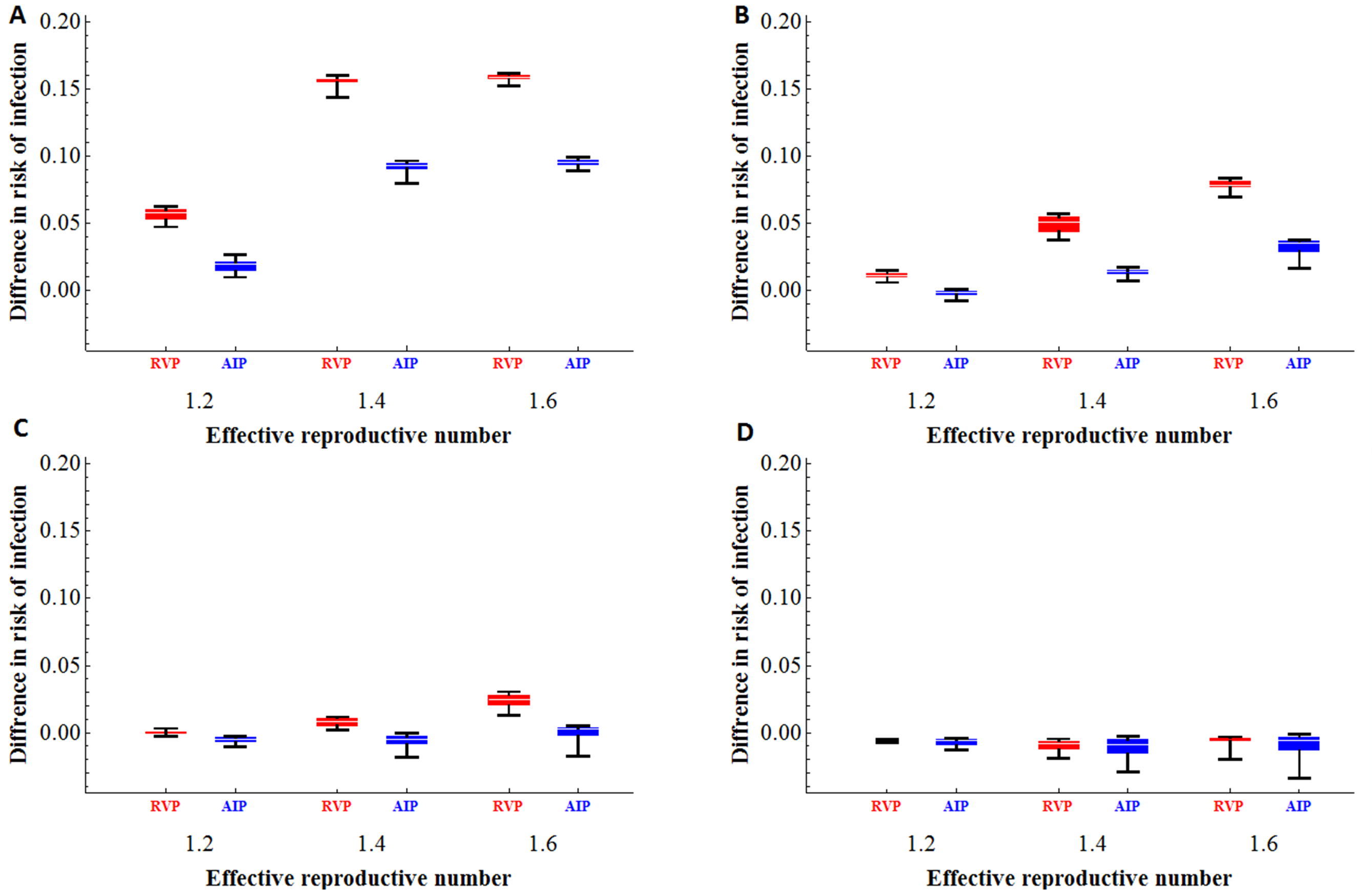

Supplement: Figure S5 — Boxplot risk of infection following vaccination. Box-and-Whisker plots of the difference between the risk of infections for RVP and PIP, and AIP and PIP, over the parameters ranges in Table 1, for cross-reactivity of A) 0%, B) 40%, C) 60%, and D) 80%, assuming vaccination coverage of 30% and vaccine efficacy of 75%. This figure corresponds to Figure 2 panels B, D, F, and G. (TIF) [file pcbi.1003643.s005.tif]

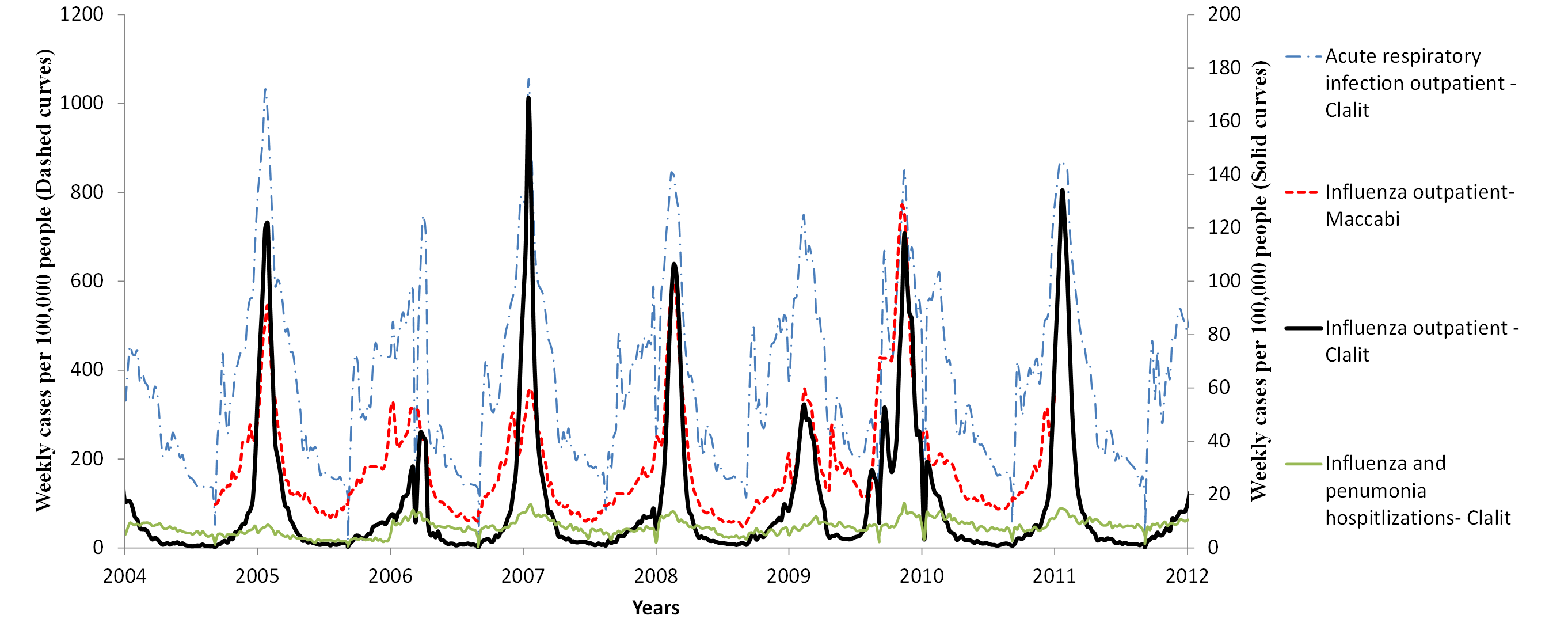

Supplement: Figure S6 — Frequency of ILI diagnosis. The right axis refers to influenza and pneumonia diagnosed in hospitals. The left axis refers to influenza diagnosis. (TIF) [file pcbi.1003643.s006.tif]
