# Supplementary material for: An Innovative Influenza Vaccination Policy: Targeting Last Season's Patients
Source: PLoS Comput Biol. 2014 May 22;10(5):e1003643. doi: 10.1371/journal.pcbi.1003643 (PMC4031061; doi:10.1371/journal.pcbi.1003643)
Supplement: Text S1 — Supporting information for contact network analysis. (DOCX) [file pcbi.1003643.s007.docx]

**Supporting information Text S1**

**An Innovative Influenza Vaccination Policy: Targeting Last Season's Patients**

**Authors:** Dan Yamin^1,2^, Arieh Gavious^2,3^, Eyal Solnik^2^, Nadav Davidovitch^4^, Ran D. Balicer^5^, Alison P. Galvani^1^, Joseph S. Pliskin^2,4,6^

**Affiliations:**

^1^Department of Epidemiology of Microbial Diseases, Yale University, 135 college St. New Haven, CT 06520

^2^Department of Industrial Engineering and Management, Ben Gurion University of the Negev, Israel

^3^Faculty of Business Administration, Ono Academic College, 104 Zahal St., Kiryat Ono 55000 Israel Israel

^4^Department of Health Systems Management, Ben Gurion University of the Negev, Israel

^5^ Clalit Research Institute, Clalit Health Services, 101 Arlozorov St., Tel Aviv, Israel

^6^Department of Health Policy and Management, Harvard School of Public Health, Boston, MA

Corresponding author: Dan Yamin, [dan.yamin@yale.edu](mailto:dan.yamin@yale.edu) , Tel. 203-600-8005.

**Text S1**

**1. Analytical framework**

The risk of future influenza infections of individuals is determined by the interplay between two countering factors: social interaction, which governs exposure probability, and cross-reactivity protection acquired from previous infection. Cross-reactivity ranges between 0-100%, where 0% corresponds to no immunological protection and 100% corresponds to full protection acquired from influenza infection in the prior year. In this section we show analytically that when no cross-reactivity is considered, last season’s patients are more likely to become infected in the succeeding season.

Consider a contact network, in which each person is represented by a node, and each contact between people is represented by an edge that connects the appropriate nodes. For simplicity assume that each individual in the population can be either central, characterized as an individual with sufficiently high connectivity, or non-central. To evaluate centrality, we calculated two common measures for each node in the network: number of contacts and K-shell (see main text). Consider a given threshold defined over centrality measure, where above this threshold the individual is considered central, and below which, the individual is considered non-central.

Let *I_i_* and *C* represent, respectively, whether an individual has been infected with influenza in season *i* and whether the individual is located in the center of the network. The risk of infection for central individuals is higher than the risk of the non-centrals [1,2]. Namely,

$P(I_{i}/C)>P(I_{i}).$ (1)

We assume that in season *i* and season *j* (*j>i*), a central individual in season *i* remains central in season *j*. Moreover, we assume that the risk of infection is independent between seasons. Namely, we want to show that

$P(I_{j}/I_{i})>P(I_{j}).$ (2)

where *I_i_* is the event where an "individual was infected in season *i*". Therefore,

$P(I_{i})=P(I_{i}/C)P(C)+P(I_{i}/\overline{C})P(\overline{C})$ , (3)

$P(I_{j})=P(I_{j}/C)P(C)+P(I_{j}/\overline{C})P(\overline{C})$ , (4)

$P(I_{j}/I_{i})=\frac{P(I_{j}\cap I{}_{i})}{P(I_{i})}=\frac{P(I_{j}\cap I_{i}/C)P(C)+P(I_{j}\cap I_{i}/\overline{C})P(\overline{C})}{P(I_{i}/C)P(C)+P(I_{i}/\overline{C})P(\overline{C})}.$ (5)

where $\overline{C}$ is the complementary event of *C*. Substituting equations (3), (4) and (5) into (2) and rearranging terms, shows that (2) holds if and only if (6) holds

 (6)

From (1) it is derived that, and thus (2) holds.

When the transmission rate between two neighbors is sufficiently lo, a policy that targets last season’s patients is equivalent to targeting a random contact of a random contact, which has been shown to be an effective immunization strategy[1].

2. Contact network simulations

## 2.1 Extension on methods in the contact network simulation section

***Estimation of the transmission rate***

We used the Susceptible-Infectious-Recovered (SIR) compartmental model [3], to evaluate disease spread within the network. According to the network configuration, an individual may infect only susceptible neighbors (i.e., nodes with whom they have edges). A cardinal parameter in our analysis is the transmission rate, defined as the mean probability per day of an infected individual to infect a specific neighbor. The transmission rate, the infectious period, and the initial proportion of susceptibles as well as the network configuration will determine the effective reproductive ratio. It is defined as the average number of secondary infective people resulting from each infective person in any specified population [3].

Thus, for a given network in each initial proportion of the susceptibility rate that represents influenza, we fitted numerically the transmission rate that would yield values of the effective reproductive number in the range presented in Table 1. This was achieved by infecting randomly 0.1% of the nodes in each network tested. Then, we simulated the first ten days in the season and marked the second generation of people infected. To prevent random noise, we performed this calculation for 1,000 iterations and verified that the mean error was less than 0.01.

## 2.2 Effect of previous illness on future infection

The value of cross-reactivity, θ, reduces the susceptibility rate in the succeeding season by $[S^{i}(0)-S^{i}(\infty))]\theta$ where $S^{i}(t)$ represents the susceptibility rate in season 1 at time t. Our simulations were implemented such that at the end of the second season, an individual that belongs to the recovered compartment moved to the susceptible compartment with probability 1-$\theta$ and stayed in the recovered compartment with probability$\theta$. To determine the risk of individuals infected in the season prior, we ran one million computerized simulations, drawing parameter values from distributions that span a biologically realistic range (Table 1), as well as different vaccination rates and efficacies in the two seasons. We evaluated the mean and 95% confidence interval of the relative risk of infection for those individuals previously infected versus individuals not previously infected (Figure S3).

**References**

1. Cohen R, Havlin S, ben-Avraham D (2003) Efficient Immunization Strategies for Computer Networks and Populations. Phys Rev Lett 91: 2–5. Available: http://link.aps.org/doi/10.1103/PhysRevLett.91.247901. Accessed 6 November 2012.

2. Christakis N a, Fowler JH (2010) Social network sensors for early detection of contagious outbreaks. PLoS One 5: e12948. Available: http://www.pubmedcentral.nih.gov/articlerender.fcgi?artid=2939797&tool=pmcentrez&rendertype=abstract. Accessed 7 November 2012.

3. Vynnycky E, White R (2010) An Introduction to Infectious Disease Modelling. Oxford University Press, USA.

4. Katriel G, Stone L (2010) Pandemic dynamics and the breakdown of herd immunity. PLoS One 5: e9565. Available: http://www.pubmedcentral.nih.gov/articlerender.fcgi?artid=2837721&tool=pmcentrez&rendertype=abstract. Accessed 30 October 2012.

5. MCCAW JM, MCVERNON J, MCBRYDE ES, MATHEWS JD (2009) Influenza :Accounting for Prior Immunity. Science (80- ) 325: 1071–1073.

6. Barnea O, Yaari R, Katriel G, Stone L (2011) Modelling seasonal influenza in Israel. Math Biosci Eng 8: 561–573. Available: http://www.ncbi.nlm.nih.gov/pubmed/21631146. Accessed 30 October 2012.

7. Watts DJ, Strogatz SH (1998) Collective dynamics of “small-world” networks. Nature 393: 440–442. Available: http://www.nature.com/nature/journal/v393/n6684/pdf/393440a0.pdf. Accessed 5 November 2012.

8. Cho E, Myers SA, Leskovec J (2011) Friendship and Mobility : User Movement In Location-Based Social Networks. KDD’11.

9. Barabasi A-L (2002) Linked: The New Science of Networks. Perseus Books. Available: http://cs.stanford.edu/people/jure/pubs/mobile-kdd11.pdf. Accessed 26 December 2012.

10. Synthetic Data Products for Societal Infrastructures and Proto-Populations: Data Set 1.0 (n.d.). Available: http://ndssl.vbi.vt.edu/Publications/ndssl-tr-06-006.pdf. Accessed 26 December 2012.
