# Supplementary material for: An Innovative Influenza Vaccination Policy: Targeting Last Season's Patients
Source: PLoS Comput Biol. 2014 May 22;10(5):e1003643. doi: 10.1371/journal.pcbi.1003643 (PMC4031061; doi:10.1371/journal.pcbi.1003643)
Supplement: Text S2 — Supporting information for data analysis. (DOCX) [file pcbi.1003643.s008.docx]

**Supporting information Text S2**

**An Innovative Influenza Vaccination Policy: Targeting Last Season's Patients**

**Authors:** Dan Yamin^1,2^, Arieh Gavious^2,3^, Eyal Solnik^2^, Nadav Davidovitch^4^, Ran D. Balicer^5^, Alison P. Galvani^1^, Joseph S. Pliskin^2,4,6^

**Affiliations:**

^1^Department of Epidemiology of Microbial Diseases, Yale University, 135 college St. New Haven, CT 06520

^2^Department of Industrial Engineering and Management, Ben Gurion University of the Negev, Israel

^3^Faculty of Business Administration, Ono Academic College, 104 Zahal St., Kiryat Ono 55000 Israel Israel

^4^Department of Health Systems Management, Ben Gurion University of the Negev, Israel

^5^ Clalit Research Institute, Clalit Health Services, 101 Arlozorov St., Tel Aviv, Israel

^6^Department of Health Policy and Management, Harvard School of Public Health, Boston, MA

Corresponding author: Dan Yamin, [dan.yamin@yale.edu](mailto:dan.yamin@yale.edu) , Tel. 203-600-8005.

**1. Influenza-like-illness case definition**

Our primary data were provided by 'Clalit Health Services’ and 'Maccabi Health Services' and are representative of the Israeli population, demographically, socio-economically and ethnically [1]. Clalit is the largest HMO in Israel, with membership varying between 3.47-3.72 million, and constituting about 53% of the Israeli population during 2003-2012 the study period. The data are based on family physician clinical diagnoses rather than on laboratory diagnosis and are likely underreported as not all influenza patients seek healthcare treatment. Here, we detail our case definition for influenza-like-illness (ILI) from both HMOs.

Clalit physicians diagnose influenza according to the ICD9 protocol (codes 487 for 'influenza' and 465 for 'acute upper respiratory infections of multiple or unspecified sites'). We also included hospitalizations due to influenza or pneumonia (ICD9, 486 ‘pneumonia organism unspecified’ as well as codes 487, 465). In the Maccabi dataset, we included influenza cases diagnosed according to their ICD10 protocol (‘influenza’ code J11).

Due to the different case definitions of influenza diagnosis between Clalit (ICD9, code 487) and Maccabi (ICD10, code J11), reported incidences vary. In particular, the case definition of influenza in the ICD9 protocol is more conservative than the one used in the ICD10. Instead of being diagnosed with influenza as codes 487, clinical studies of laboratory-confirmed tests worldwide [2] and in Calilt [3] suggest that during the influenza season, the majority of individuals seeking medical care for acute upper respiratory infections (AURI) are infected with influenza. In addition, an acute upper respiratory infection has relatively similar clinical symptoms as influenza, and its progression pattern along the season highly correlates with influenza progression (See Figure S6, and Table S1). Therefore, for the Clalit patients, we considered an AURI as case of ILI (Table S2).

Pneumonia is the primary complication of influenza infection and, as such, accounts for the majority of influenza hospitalizations [4]. Even when pneumonia is caused by bacteria and respiratory viruses other than influenza, inﬂuenza has been shown to increase the susceptibility for other etiologies of pneumonia [5,6]. Consequently, as interventions that effectively reduce influenza will concomitantly impact pneumonia arising either directly from influenza or indirectly from bacterial etiologies [6,7], we included hospitalization due to pneumonia as well (Table S2).

To avoid biases derived from the effect of birth, death, or switching HMOs, we excluded individuals who had not been members during both calendar years of the two seasons tested. Conservatively, we also excluded members who had more than four visits in a given season. In the Clalit dataset, to prevent double counting we considered an individual as a patient diagnosed in clinics only if not hospitalized in the same season.

Influenza is highly seasonal with a peak in the winter. Weekly sampling of laboratory sero-prevalence tests by the Israeli Center for Disease Control during the influenza season (broadly defined from September 1 to April 1) suggests between 35-95% of the cases diagnosed as influenza are accurately confirmed by the sero-prevalence test around the seasonal peak [3]. Outside of the influenza season, most influenza diagnoses are incorrect [3]. Consequently, we focused on the data from September 1 to April 1 in our analysis and excluded data outside of these dates.

The total Clalit data set includes about 470,000 records of influenza diagnosed in clinics and 8.1 million records of AURI, and 164,000 records of hospitalization due to influenza or pneumonia. The Maccabi data set includes 380,000 records of outpatient influenza. Both data sets cover complete influenza seasons, three of which were dominated by influenza B and the rest dominated by influenza A, including subtype H1N1 introduced in 2009-2010.

**Table S1: Influenza and AURI correlation matrix for spread of disease**

|  |  | Outpatient  Influenza (Maccabi) | Outpatient AURI (Clalit) | Outpatient Influenza (Clalit) | Hospitalizations Influenza and pneumonia  (Clalit) |
| --- | --- | --- | --- | --- | --- |
| Outpatient  Influenza (Maccabi) | **Pearson Correlation**  **N** | 1  418 | 0.756  418 | 0.814  418 | 0.484  418 |
| Outpatient AURI (Clalit) | **Pearson Correlation**  **N** | 0.756  418 | 1  472 | 0.834  472 | 0.611  472 |
| Outpatient Influenza (Clalit) | **Pearson Correlation**  **N** | 0.814  418 | 0.834  472 | 1  472 | 0.57  472 |
| Hospitalizations influenza and pneumonia  (Clalit) | **Pearson Correlation**  **N** | 0.484  418 | 0.611  472 | 0.57  472 | 1  472 |

*All numbers are significant at level of 0.01

**2. The relative risk of infection**

For the evaluation of the relative risk given infection in the prior season, we defined the following events:

$I_{i}^{O}$ – A member in the health maintenance organization in both seasons *i* and *i+*1, and is outpatient with ILI at least once in the season *i*. The relative risk for an individual previously infected *i* to become outpatient with ILI in subsequent year compared with individuals not infected, $RR^{O,O}$was evaluated as follows:

$RR^{O,O}=\frac{\frac{|I_{i}^{o}\cap I_{i+1}^{o}|}{|I_{i}^{o}|}}{\frac{|I_{i+1}^{o}\text{ }\cap\overline{I_{i}^{o}|}}{\overline{|I_{i}}|}}$ (1),

where $\overline{A}$ represents the complement of event *A*, and *|A|* represents the number of members in event *A*. We likewise evaluated $RR^{O,H}$, the relative risks of individual outpatients with ILI to become hospitalized with ILI in the subsequent year, $RR^{H,O}$, the relative risks of individuals hospitalized with ILI to become outpatient with ILI in the subsequent year, and $RR^{H,H}$, the relative risk of individuals hospitalized to become hospitalized in the subsequent year.

Not all influenza patients seek medical treatment [1,27]**,** and some individuals might have higher tendency to seek medical treatment when infected with influenza than others, potentially leading to an overestimation of relative risks. In addition, some individuals are more likely to visit the same physician when infected with influenza, and the latter might not diagnosis the infection as ILI. Thus, we compared evaluations of the policies under the conservative assumption that individuals can be divided into those that either always seek medical treatment when infected, or never seek medical treatment. Under this assumption, we calculated an adjusted relative risk of outpatient infection by disregarding members who were never an outpatient within the entire period tested (2003-2012 in Clalit dataset and 1998-2010 in Maccabi dataset). Affectively, this reduces the total number of individuals not infected, $\overline{|I_{i}}|$in (1).

**Table S2: Influenza-like-illness case definition**

| Clinical diagnosis | Health maintenance organization | Influenza like illness (ILI) | References |
| --- | --- | --- | --- |
| GP visits | **Maccabi Health Services** | Influenza due to unidentified influenza virus (J11- ICD 10 code) diagnosed between September 1 to April 1 | [3] |
|  | **Clalit Health Services** | 1. Influenza (487- ICD 9 code) diagnosed between September 1 to April 1 and not hospitalized with influenza  2. Acute upper respiratory infections of multiple or unspecified sites (AURI) (465- ICD 9 code) diagnosed between September 1 to April 1 and not hospitalized with influenza | [2,3] |
| Hospitalizations | **Clalit Health Services** | 1. Influenza (487- ICD 9 code)  2. Pneumonia (ICD9, as code 486 and 481-484) diagnosed between September 1 to April 1. | [6,7,10–12] |

**References**

1. Memebership in HMO’s (n.d.). Natl Insur Inst Isr. Available: http://www.btl.gov.il/Publications/survey/Pages/saker233.aspx.

2. WHO | Influenza (2003). World Heal Organ. Available: http://www.who.int/mediacentre/factsheets/2003/fs211/en/. Accessed 1 January 2013.

3. Influenza monitoring in Israel and the World (2012). Isr CDC. Available: http://www.old.health.gov.il/pages/default.asp?PageId=3987&parentId=507&catId=78&maincat=25. Accessed 18 November 2012.

4. Rudan I, Boschi-Pinto C, Biloglav Z, Mulholland K, Campbell H (2008) Epidemiology and etiology of childhood pneumonia. Bull World Health Organ 86: 408–416. Available: http://www.pubmedcentral.nih.gov/articlerender.fcgi?artid=2647437&tool=pmcentrez&rendertype=abstract. Accessed 24 January 2014.

5. Shrestha S, Foxman B, Weinberger DM, Steiner C, Viboud C, et al. (2013) Identifying the Interaction Between Influenza and Pneumococcal Pneumonia Using Incidence Data. Sci Transl Med 5: 191ra84–191ra84. Available: http://stm.sciencemag.org/cgi/doi/10.1126/scitranslmed.3005982. Accessed 27 June 2013.

6. Grohskopf L, Shay DK, Shimabukuro T, Sokolow L, Keitel W, et al. (2013) Prevention and Control of Seasonal Influenza with Vaccines: Recommendations of the Advisory Committee on Immunization Practices — United States, 2013–2014. Available: http://www.cdc.gov/mmwr/preview/mmwrhtml/rr6207a1.htm?s_cid=rr6207a1_w. Accessed 5 January 2014.

7. Nichol KL, D. NJ, Nelson DB, Mullooly JP, Hak E (2007) Effectiveness of Influenza Vaccine in the Community-Dwelling Elderly. N Engl J Med 357: 1373–138. Available: http://www.nejm.org/doi/full/10.1056/NEJMoa070844. Accessed 3 May 2013.

8. Molinari N-AM, Ortega-Sanchez IR, Messonnier ML, Thompson WW, Wortley PM, et al. (2007) The annual impact of seasonal influenza in the US: measuring disease burden and costs. Vaccine 25: 5086–5096. Available: http://dx.doi.org/10.1016/j.vaccine.2007.03.046. Accessed 5 March 2013.

9. Bridges CB, Thompson WW, Meltzer MI, Reeve GR, Talamonti WJ, et al. (2000) Effectiveness and Cost-Benefit of Influenza Vaccination of Healthy Working Adults. JAMA 284: 1655. Available: http://jama.jamanetwork.com/article.aspx?articleid=193139. Accessed 28 February 2014.

10. Huss A, Scott P, Stuck AE, Trotter C, Egger M (2009) Efficacy of pneumococcal vaccination in adults: a meta-analysis. CMAJ 180: 48–58. Available: http://www.pubmedcentral.nih.gov/articlerender.fcgi?artid=2612051&tool=pmcentrez&rendertype=abstract.

11. Jackson ML, Nelson JC, Weiss NS, Neuzil KM, Barlow W, et al. (2008) Influenza vaccination and risk of community-acquired pneumonia in immunocompetent elderly people: a population-based, nested case-control study. Lancet 372: 398–405. Available: http://dx.doi.org/10.1016/S0140-6736(08)61160-5. Accessed 30 April 2013.

12. Ferdinands JM, Gargiullo P, Haber M, Moore M, Belongia EA, et al. (2013) Inactivated influenza vaccines for prevention of community-acquired pneumonia: the limits of using nonspecific outcomes in vaccine effectiveness studies. Epidemiology 24: 530–537. doi:10.1097/EDE.0b013e3182953065.
